# NLRP3 Contributes to Sarcopenia Associated to Dependency Recapitulating Inflammatory-Associated Muscle Degeneration

**DOI:** 10.3390/ijms25031439

**Published:** 2024-01-24

**Authors:** Eduardo Antuña, Yaiza Potes, Francisco Javier Baena-Huerta, Cristina Cachán-Vega, Nerea Menéndez-Coto, Eva Álvarez Darriba, Marta Fernández-Fernández, Natalie Burgos Bencosme, Manuel Bermúdez, Eva María López Álvarez, José Gutiérrez-Rodríguez, José Antonio Boga, Beatriz Caballero, Ignacio Vega-Naredo, Ana Coto-Montes, Claudia Garcia-Gonzalez

**Affiliations:** 1 Research Group OSKAR, Instituto de Investigación Sanitaria del Principado de Asturias (ISPA), 33011 Oviedo, Spain; 2 Department of Morphology and Cell Biology, University of Oviedo, 33006 Oviedo, Spain; 3 Instituto de Neurociencias del Principado de Asturias (INEUROPA), 33006 Oviedo, Spain; 4 Geriatric Service, Monte Naranco Hospital, 33012 Oviedo, Spain; 5 Grupo de Investigación Microbiología Traslacional, Instituto de Investigación Sanitaria del Principado de Asturias (ISPA), 33011 Oviedo, Spain; 6 Servicio de Microbiología, Hospital Universitario Central de Asturias (HUCA), 33011 Oviedo, Spain

**Keywords:** sarcopenia, NLRP3 inflammasome, ASC, Caspase-1, inflammaging, dependency, MYH3, SASP

## Abstract

Sarcopenia, a complex and debilitating condition characterized by progressive deterioration of skeletal muscle, is the primary cause of age-associated disability and significantly impacts healthspan in elderly patients. Despite its prevalence among the aging population, the underlying molecular mechanisms are still under investigation. The NLRP3 inflammasome is crucial in the innate immune response and has a significant impact on diseases related to inflammation and aging. Here, we investigated the expression of the NLRP3 inflammasome pathway and pro-inflammatory cytokines in skeletal muscle and peripheral blood of dependent and independent patients who underwent hip surgery. Patients were categorized into independent and dependent individuals based on their Barthel Index. The expression of NLRP3 inflammasome components was significantly upregulated in sarcopenic muscle from dependent patients, accompanied by higher levels of Caspase-1, IL-1β and IL-6. Among older dependent individuals with sarcopenia, there was a significant increase in the MYH3/MYH2 ratio, indicating a transcriptional shift in expression from mature to developmental myosin isoforms. Creatine kinase levels and senescence markers were also higher in dependent patients, altogether resembling dystrophic diseases and indicating muscle degeneration. In summary, we present evidence for the involvement of the NLRP3/ASC/NEK7/Caspase-1 inflammasome pathway with activation of pro-inflammatory SASP in the outcome of sarcopenia in the elderly.

## 1. Introduction

Improving healthspan, known as the period of life spent in good health devoid of age-related chronic illnesses and disabilities, and not only lifespan has become a new challenge for our society. Achieving this goal necessitates a better understanding of the molecular mechanisms regulating aging and healthy aging, together with a comprehensive study of the involved factors and their interactions. The postponement of age-related declines and the enhancement of functional measures associated with aging are both crucial to facilitate improved diagnosis of pathologies, while also aiding in their prevention and treatment.

Sarcopenia, a condition characterized by loss of muscle mass and mobility function resulting from a deterioration of skeletal muscle quality, stands as a key factor that significantly impacts healthspan and contributes to frailty [1]. This condition progressively evolves and is associated with a risk of adverse outcomes such as physical disability, poor life quality, and death [2]. Moreover, sarcopenia is also associated with a higher risk of hospitalization, worse hospitalization recovery, decreased discharge rate, and increased level of dependency after hospitalization [3]. The multifactorial nature of sarcopenia underscores the importance of adopting a comprehensive perspective in order to gain an integrated understanding of the interplay between its various contributing factors.

The use of biomarkers for studying sarcopenia can appropriately provide clinically relevant information to improve the diagnosis, prognosis, and therapeutics [4,5]. The process of aging is marked by the occurrence of systemic chronic inflammation, which is accompanied by cellular senescence, immunosenescence, organ dysfunction, and age-related diseases [6]. Inflammatory markers have been associated with loss of both muscle strength and mass [7,8]. However, the complexity of the immune system necessitates a more thorough evaluation of numerous factors, particularly in human studies.

Inflammation is a double-edged sword that has both protective and harmful effects under various pathological conditions. While necessary to trigger regeneration and repair, a chronic activation of the inflammatory pathways is associated with disease [9,10]. During the process of aging, various forms of damage accumulation occur at the molecular, cellular, and tissue levels. In response to injury, death, or exposure to stressors, endogenous molecules are secreted or exposed by dying or injured cells. These molecules, known as damage-associated molecule patterns (DAMPs), serve as alarm signals to activate the immune system in response to tissue damage or injury, thereby facilitating a wound-healing response [11]. These include reactive oxygen species (ROS) released from damaged mitochondria, extracellular nucleotides like ATP, high mobility group box (HMGB) 1 protein, oxidized low density lipoprotein, amyloid-beta (Aβ), islet amyloid polypeptide, and particulates like monosodium urate (MSU) crystals and cholesterol crystals [12]. DAMPs can be recognized by common pattern recognition receptors, which then initiate an immune and inflammatory response.

Inflammaging, a term used to describe age-related inflammation, is primarily driven by a process known as sterile inflammation and can be triggered by DAMP accumulation [13]. Inflammasomes, which are multiprotein oligomers found in the cytosol of cells, play a key role in initiating inflammatory responses by activating Caspase-1. Among the different inflammasomes, the NLRP3 is most studied and characterized due to its ability to respond to a wide range of stimuli and has been implicated in the pathogenesis of several autoinflammatory diseases and age-related inflammatory diseases [14,15].

Several reports have linked activation of the NLRP3 inflammasome to cardiovascular disease. In patients with dilated cardiomyopathy (DCM), the myocardial tissues have shown hyperactivated NLRP3 inflammasome, which leads to pyroptotic cell death through Caspase-1 activation in cardiomyocytes [16]. Conversely, inhibiting the NLRP3 inflammasome has demonstrated a protective effect against atrial fibrillation caused by cardiovascular aging [17], as well as overload-induced cardiac hypertrophy [18]. These findings suggest that the NLRP3 inflammasome is a promising therapeutic target not only for heart failure [19], but also for other muscle conditions such as sarcopenia.

NLRP3 involvement in skeletal muscle has been reported in muscle atrophy. In the context of sepsis-induced skeletal muscle atrophy, the use of NLRP3 inhibitors has shown promising effects in alleviating the condition by inhibiting catabolic processes and cachexia-associated inflammation [20]. Activation of the NLRP3 inflammasome following denervation has been found to induce pyroptosis and to upregulate genes associated with muscle atrophy, thereby facilitating the activation of the ubiquitin-proteasome system (UPS) responsible for muscle proteolysis. Conversely, studies conducted on NLRP3 knockout mice have demonstrated that reduced IL-1β levels due to NLRP3 deficiency mitigate sepsis-induced skeletal muscle atrophy [21]. NLRP3 knockout in skeletal muscle ameliorates atrophy and inhibition of NLRP3 in C2C12 myotubes results in reduced pyroptosis and atrophy in vitro [22]. Additionally, NLRP3 inflammasome activation may modulate insulin-mediated signaling in skeletal muscle during insulin resistance conditions [23]. However, the role of the NLRP3 inflammasome in human sarcopenia remains poorly understood.

The gradual degeneration of skeletal muscle caused by sarcopenia has the potential to induce, at early stages, frailty in the elderly population. Moreover, it can be regarded as a primary contributor to physical frailty and may even serve as an early indication of this state. According to the recommendations of the European Working Group on Sarcopenia in Older People (EWGSOP), sarcopenia can be categorized in pre-sarcopenia stage, characterized by low muscle mass with no impact on muscle strength or physical performance, whereas the second stage, sarcopenia, is characterized by low muscle mass along with either low muscle strength or low physical performance [24]. In clinical practices, the identification of sarcopenia holds significant value. Researchers have dedicated their efforts to investigating different methods and tools that can be employed to effectively recognize this condition and evaluate frailty in sarcopenic patients. The Barthel Index (BI) is a widely recognized measure that is commonly employed to broadly characterize the dependency status of individuals [25,26]. The Barthel Index for Activities of Daily Living is a scale that assesses an individual’s capacity to perform everyday tasks. Originally developed by Mahoney and Barthel in 1965 [27], it was later modified by Granger, Dewis, Peters, Sherwood, and Barrett in 1979 [28]. The modification involved assigning 0–10 points for each item, resulting in a total possible score ranging from 0 to 100. A score of 0 indicates complete dependence, while a score of 100 indicates complete independence. The Barthel Index is commonly used to evaluate physical functional dependency and has been found to be significantly associated with the risk of sarcopenia and frailty [29,30]. The recent update to the EWGSOP algorithm for screening sarcopenia incorporates various components such as the Simple Questionnaire to Rapidly Diagnose Sarcopenia (SARC-F) questionnaire, the assessment of muscle strength, muscle quantity or quality, and the evaluation of physical performance. This algorithm has identified that BI can serve as a predictive factor to assist healthcare professionals in the early detection of sarcopenia and in determining appropriate therapeutic interventions [31]. Additionally, the BI has been found to be correlated with Grip Strength and Physical Activity Level [32], as well as with low skeletal muscle index [33] and gait speed [34]. At the molecular level, a lower BI is associated with muscle decline, characterized by a reduction in the expression of myogenic regulatory factors and satellite cell markers, an increase in myostatin levels, and an increase in apoptosis [30]. Consequently, we utilize the BI as the foundation for classifying patients into different categories of frailty, dependency, and sarcopenia severity. Henceforth, patients with a BI score below 70 will be referred to as dependent patients (DP), while those with a BI score of 100 will be classified as independent patients (IP).

Here, we investigated the role of NLRP3 in human skeletal muscle, focusing on its differential activation in dependent and independent patients. The objective was to gain a comprehensive understanding of the interplay between this condition and the molecular implications associated with it.

## 2. Results

### 2.1. Study Sample Characteristics

Table S3 indicates that there are no differences in the distribution of blood cell types, including each type of white blood cell (WBC): Neutrophils, Lymphocytes, Monocytes, Eosinophils and Basophils, as well as red blood cells (RBC), between independent (IP) and dependent patients (DP).

### 2.2. NLRP3 Inflammatory Pathway Is Upregulated in Sarcopenic Muscle from DP

**Figure 1:**
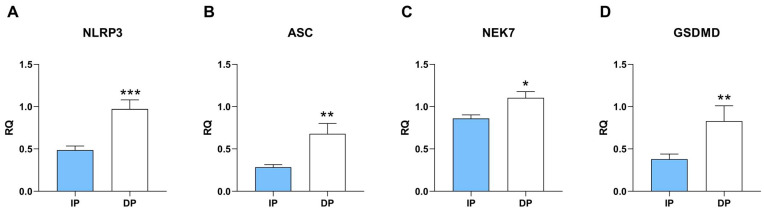
Higher expression of the components of NLRP3 pathway in muscle samples from dependent vs. independent patients. RT-PCR analysis of (**A**) NLRP3, (**B**) ASC, (**C**) NEK7, (**D**) GSDMD relative quantification (RQ) to HPRT1 (n = 30 for each of the two groups). * *p* ≤ 0.05, ** *p* ≤ 0.01, *** *p* ≤ 0.001.

In order to investigate the involvement of the NLRP3 inflammasome pathway in human sarcopenic muscle, we first examined gene expression of the NLRP3 components and effectors. We found higher mRNA expression of the main components *NLRP3* and *ASC* in dependent sarcopenic muscle compared to independent (Figure 1A,B). Moreover, we found that dependent muscle exhibits higher mRNA expression of the *NEK7* kinase (Figure 1C), a key regulator of the oligomerization and assembly of the NLRP3 inflammasome, indicating increased activation of this pathway in sarcopenic muscle from DP. In turn, in skeletal muscle from dependent patients, we found upregulation of the mRNA expression of gasdermin D (*GSDMD*) (Figure 1D), the mediator of inflammasome-dependent pyroptosis and downstream inflammation. These results indicate that NLRP3 inflammasome activation is enhanced in human sarcopenic muscle from DP compared to independent muscle.

### 2.3. Activation of Inflammatory Caspase-1 and Secretion of Cytokines

**Figure 2:**
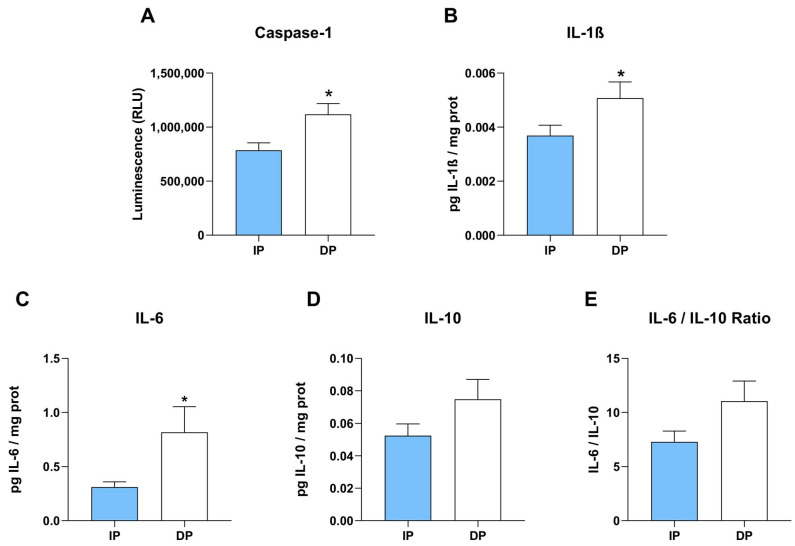
Higher interleukin expression in plasma samples from dependent patients vs. independent patients. (**A**) Caspase-Glo^®^ 1 Inflammasome Assay in muscle lysates evaluated by bioluminescence (n = 25 for each of the two groups). (**B**–**D**) Expression of plasma cytokines measured as pg IL/ mg total protein (**B**) IL-1β (n = 19/19), (**C**) IL-6 (n = 19/20), (**D**) IL-10 (n = 20/19), and (**E**) IL-6/IL-10 ratio (n = 19/19). * *p* ≤ 0.05.

Upon NLRP3 inflammasome priming, activated Caspase-1 triggers proteolytic cleavage of pro-IL-1β and gasdermin D (GSDMD), leading to pyroptosis. To confirm if the observed increase in NLRP3 mRNA expression is associated with higher expression of Caspase-1 in sarcopenic muscle of dependent patients, we measured Caspase-1 activity by bioluminescence assay. Strikingly, we found a significant increase in Caspase-1 activity in muscle lysates from sarcopenic muscle from DP compared to sarcopenic muscle from IP (Figure 2A).

To further confirm the activation of the inflammasome pathway upon dependency, the plasma levels of human IL-1β were measured by ELISA (Figure 2B). Intriguingly, we found that dependent patients display significantly higher plasma levels of IL-1β than independent patients, indicating a higher IL-1β production dependent on Caspase-1. Additionally, circulating levels of IL-6 and IL-10, two sarcopenia-related cytokines, were measured by ELISA. We found that the pro-inflammatory environment we observed in sarcopenic muscle from dependent patients is associated with a significant increase in IL-6 plasma levels (Figure 2C). IL-6 is a very well-known pro-inflammatory cytokine closely related to skeletal muscle weakness in the elderly with sarcopenia [35]. IL-10, conventionally recognized as an anti-inflammatory cytokine, exerts pleiotropic effects recently related to immune system responses, but has an unclear role in sarcopenia [9,36]. IL-10 circulating levels were higher in sarcopenic muscle from DP, although no significant differences were found compared to independent patients (Figure 2D). Moreover, we did not find significant differences in the IL-6/IL-10 ratio between independent and dependent patients (Figure 2E). Higher levels of IL-6 and IL-10, both indicators of sarcopenia and muscle strength loss, underlay the ongoing inflammatory process compromising muscle quality in dependent patients.

### 2.4. Skeletal Muscle from DP Recapitulates Muscle Dystrophy

**Figure 3:**
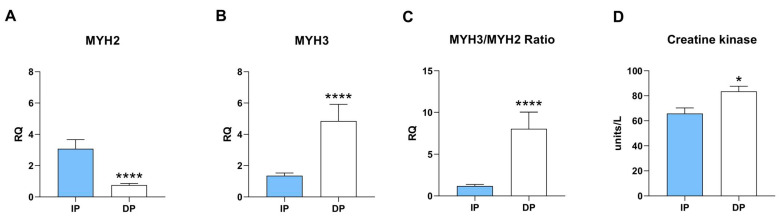
Increased muscle atrophy in dependent muscle. RT-PCR analysis of (**A**) MYH2, (**B**) MYH3, (**C**) ratio MYH3/MYH2 (n = 30 for each of the two groups); (**D**) creatine kinase activity in protein lysates from independent- and dependent-skeletal muscle lysates (n = 16 for each of the two groups). * *p* ≤ 0.05, **** *p* ≤ 0.0001.

Duchenne muscular dystrophy (DMD) is a fatal muscle disorder caused by the lack of the dystrophin protein, which is characterized by cycles of degeneration and regeneration leading to irreversible muscle degeneration and fibrosis [37]. One of the hallmarks of DMD is the increased expression of developmental myosin isoforms, such as embryonic myosin (MYH3). We wanted to assess if sarcopenic muscle from dependent patients also exhibits alterations in myosin isoform expression. We evaluated by RT-PCR the expression levels of *MYH3* and of the adult fast isoform *MYH2*. We found higher expression of *MYH3* together with decreased expression of *MYH2* in sarcopenic muscle from DP (Figure 3A,B), leading to a highly significant increase in the *MYH3*/*MYH2* ratio in dependent muscle (Figure 3C). These results indicate transcriptional dysregulation and aberrant expression of development of myosin isoforms in dependent muscle, which resembles the features of muscular dystrophy.

We thus investigated the activation of muscle creatine kinase (CK), which serves as a marker of muscle damage and as an indicator of DMD in patients and mouse models [38,39]. Importantly, CK activity was significantly higher in dependent muscle compared to independent, evidencing a more severe stage of skeletal muscle damage (Figure 3D).

These findings point to the degenerative and pro-inflammatory environment of the sarcopenic muscle of dependent patients, which partially recapitulates the features of DMD and is associated with atrophy and muscle weakness.

### 2.5. Increase of Infiltrated Immune Cells in Sarcopenic Muscle from DP

**Figure 4:**
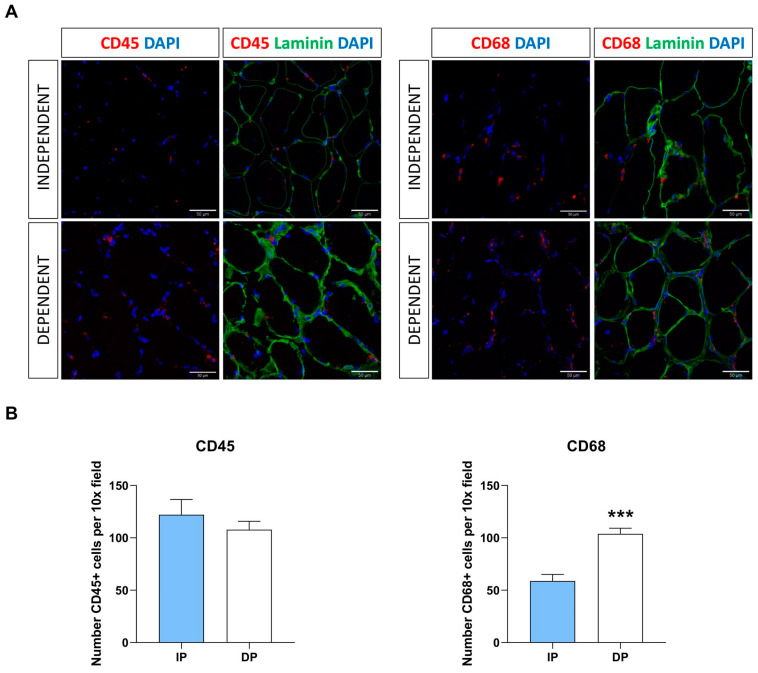
Increase in infiltrated immune cells in sarcopenic muscle from DP. (**A**) Immunofluorescence staining of skeletal muscle sections from independent- and dependent patients for CD45 and CD68. (**B**) Quantification of CD45^+^- and CD68^+^-cells in independent and dependent patients (n = 5 for each of the two groups)**.** *** *p* ≤ 0.001.

In light of the observed pro-inflammatory milieu characterizing sarcopenic muscle from DP, we assessed the presence of inflammatory cells by histological analysis. Strikingly, we found a significant increase in the numbers of macrophages (CD68^+^) but not of leucocytes (CD45^+^) in sarcopenic muscle from DP compared to IP (Figure 4A,B). Tissue-infiltrated immune cells are associated with a higher number of circulating concentrations of proinflammatory cytokines IL-1β and IL-6, indicating that NLRP3 activation in sarcopenic skeletal muscle drives recruitment of immune cells. These results suggest that the NLRP3 inflammasome plays a key role in immune cell infiltration of sarcopenic muscle from DP.

### 2.6. NLRP3 Induces Cellular Senescence, Necroptosis, and Increases DNA Damage

**Figure 5:**
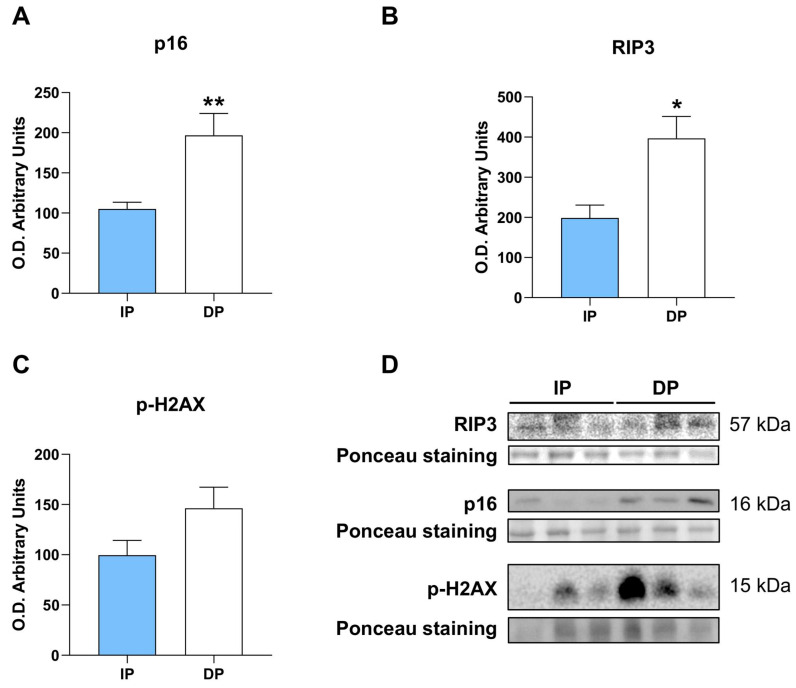
NLRP3 induces cellular senescence in dependent muscle. Immunoblots and quantification of (**A**) p16, (**B**) RIP3, (**C**) p-H2AX in muscle lysates from independent- and dependent patients, (**D**) Western blotting representative images (n = 30 for each of the two groups). * *p* ≤ 0.05, ** *p* ≤ 0.01.

We next evaluated whether NLRP3 inflammasome activation can trigger cellular senescence in skeletal muscle. We first measured the expression of the senescence-associated protein p16^INK4a^ which was higher in sarcopenia in sarcopenic muscle from DP (Figure 5A).

The receptor-interacting protein kinase 3 (RIP3/RIPK3) is a critical regulator of a type of programmed cell death called necroptosis [40]. Necroptosis is an inflammatory form of cell death associated with sterile inflammation. RIP3 directly interacts with NLRP3 to form RIP3-NLRP3 complexes and lead to Caspase-1 activation, resulting in interleukin production in lung injury [41]. To study the potential involvement of RIP3 in the activation of NLRP3 underlying sarcopenia in dependent patients, we examined the levels of RIP3 protein expression in skeletal muscle lysates by Western blot analysis. Importantly, we found significantly higher expression of RIP3 in sarcopenic muscle from DP, indicating that RIP3 may participate in the activation of NLRP3 in DP (Figure 5B).

Moreover, DNA damage response was analyzed and revealed higher although no significant levels of H2AX phosphorylation in sarcopenic muscle from DP (Figure 5C), suggesting higher oxidative stress and DNA damage, and corroborating the increase in cellular senescence upon severe skeletal muscle loss.

Collectively, these data show that NLRP3 promotes activation of the p16-mediated senescence pathway and RIP3 necroptotic signaling, and that DNA damage may participate in sustaining pro-inflammatory and degenerative pathways via the SASP.

### 2.7. Damage-Associated Pattern

**Figure 6:**
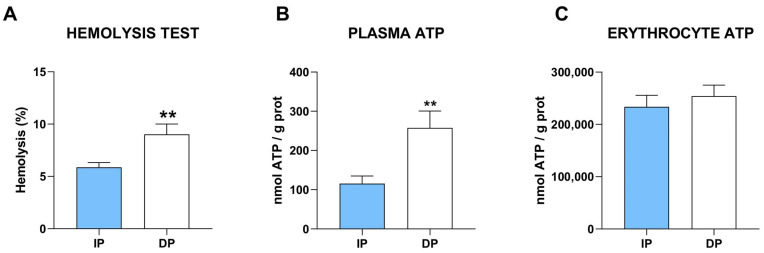
Higher damage-associated molecules in dependent muscle. (**A**) Hemolysis test expressed in hemolysis percentage (%) (n = 30 for each of the two groups); (**B**,**C**) ATP levels in (**B**) plasma (n = 17 for each of the two groups) and (**C**) erythrocytes (n = 22 for each of the two groups) of independent- and dependent patients. ** *p* ≤ 0.01.

To investigate the potential sources of inflammation, we evaluated two main types of damage-associated molecules potentially involved in NLRP3 activation. Hemolysis represents a major inflammatory mechanism that is associated with inflammatory patterns. Erythrocyte hemolysis results in the release of large quantities of red cell damage-associated molecular patterns with high inflammatory potential [42]. To evaluate the erythrocyte resistance to oxidative stress, we performed a hemolysis test. Results revealed that erythrocytes from dependent individuals show a higher degree of hemolysis compared to erythrocytes obtained from independent individuals (Figure 6A), indicating a higher susceptibility to cell damage by oxidative stress.

Among others, one of the molecules leading to NLRP3 activation is adenosine triphosphate (ATP), a well-known activator of the pro-inflammatory environment via activation of Caspase-1 [43]. Extracellular ATP is to be considered a tissue damage-associated molecule that is potentially released from damaged cells during tissue destruction [44]. We thus evaluated the free ATP levels in plasma from dependent and independent patients and found a significant increase in plasma ATP in dependent patients (Figure 6B). Interestingly, erythrocyte levels of ATP showed no significant differences between groups (Figure 6C), indicating that the higher levels of ATP are restricted to the acellular blood fraction. These data suggest that the release of ATP from damaged cells serves as a damage-associated molecule activating the NLRP3 inflammatory pathway in dependent patients.

## 3. Discussion

Skeletal muscle regeneration depends to a large extent on innate immune responses. While after injury, the activation of the immune response and the recruitment of inflammatory cells is essential in the process of muscle regeneration and repair [45], the systemic, chronic, sterile, and low-grade inflammation known as inflammaging exhibits opposite effects [46]. These opposite effects of the immune system reveal the relevance of a fine-tuning of the immune response and the necessity to study the implications in health and disease.

Sterile inflammation occurs in the absence of pathogens and is due to the recognition by the immune system of damage-associated stimuli. Some of the most studied cases of the pathogen-free inflammation are ischemic stroke [47] and heart ischemia-reperfusion [48]. In response to these events, massive cell death causes the release of DAMPs contributing to the induction of the immune response. Furthermore, administration of neutralizing HMGB1 antibodies reduces damage in reperfused hearts [49] and attenuates ischemic brain damage by inhibiting the extravasation of proteins causing brain edema [50].

Sterile inflammation is central to many age-associated diseases and sarcopenia is a multifactorial disease with a gradual deterioration of skeletal muscle quality and loss of muscle functionality. Based on the relevance of the inflammasome NLRP3 in elderly diseases and frailty [51], we investigated the implications of the activation of the NLRP3 inflammation pathway in human sarcopenia. We found higher expression of the NLRP3 pathway in muscle biopsies from dependent patients compared to independent patients. An increasing number of studies have demonstrated that the activation of the NLRP3 inflammasome plays a crucial role in the development and progression of inflammation-induced skeletal muscle atrophy in cellular and animal models [20,22]. The NLRP3 inflammatory pathway was already reported to be involved in cardiac remodeling during pressure overload in an NLRP3 KO mouse model and resulted in reduced sarcopenia onset and progression, loss of muscle glycolytic potential, and mitochondrial dysfunction [52,53]. Genetic disruption of NLRP3 or pharmacological inhibition both reduce skeletal muscle atrophy caused by inflammation via decreased expression of IL-1β in in vivo and in vitro models [20,21,54].

Evidence of a link between human muscle pathology and the NLRP3 inflammasome have been reported by Kummer et al. (2023) [55]. The authors studied a type of idiopathic inflammatory myositis, inclusion body myositis (IBM), characterized by inflammation and protein accumulation. They found pronounced upregulation of NLRP3 in muscle biopsies from IBM patients compared to healthy skeletal muscle, and in an in vitro model using human myoblasts and primary human myotubes. These results revealed that in myotubes, NLRP3 is regulated independently from infiltrative immune cells. In human sarcopenic muscle from DP, we found a similar upregulation of these pathways, resembling sarcopenia to IBM and demonstrating the implications of the NLRP3 inflammasome in sarcopenic muscle from DP and the inflammatory nature of this debilitating condition.

Our hypothesis that activation of NLRP3 with the course of the sarcopenic condition might enhance muscle degeneration in sarcopenic muscle from DP is supported by independent studies on muscle atrophy. Amyotrophic lateral sclerosis (ALS) is a neurodegenerative disorder that leads to the degeneration and paralysis of voluntary muscles due to the gradual deterioration of motor neurons. In a mouse model of ALS, the *SOD1G93A* transgenic mice, the NLRP3 inflammasome is upregulated in skeletal muscle. Furthermore, blood samples from ALS patients exhibited a noteworthy rise in NLRP3 transcriptional levels when compared to those of healthy controls [56].

Upon activation of NLRP3, ASC facilitates the recruitment of pro-Caspase-1, leading to the formation of an NLRP3 inflammasome. Consequently, the inflammasome has the ability to induce the activation of pro-Caspase-1. Subsequently, activated Caspase-1 cleaves pro-IL-1β, leading to the generation of mature IL-1β [57]. Most recently, NEK7, a member of the family of mammalian NIMA-related kinases (NEKs), was found to regulate NLRP3 oligomerization and activation in a potassium efflux-dependent way [58]. Some studies observed that the NEK7/NLRP3 inflammasome-signaling pathway is involved in diabetic vascular injury [59] or in inflammation-related diabetic cardiomyopathy [60], a type of sterile inflammation, with a role in oligomerization and activation of the NLRP3 inflammasome complex. In our study, we found higher expression of the NLRP3 components and an increase in Caspase-1 and IL-1β levels together with higher expression of NEK7 in sarcopenic muscle from DP. Therefore, NEK7 might be involved in the activation of the inflammasome in an NEK7/NLRP3-dependent way in sarcopenic muscle.

Gasdermins are a group of proteins that play a crucial role in the immune response by forming membrane pores. Among these proteins, gasdermin D (GSDMD) has been recognized as the key player in pyroptosis, a form of cell death, through the creation of membrane pores in Caspase-1-activating inflammasomes [61]. Gasdermin D also disrupts both the inner and outer membranes of the mitochondria, resulting in a decline in the quantity of mitochondria, initiation of mitophagy, generation of reactive oxygen species (ROS), disruption of transmembrane potential, attenuated oxidative phosphorylation (OXPHOS), and liberation of mitochondrial proteins and DNA from the matrix and intermembrane space enhancing cell death by pyroptosis [62]. A previous study has linked sarcopenic muscle from DP in the elderly to mitochondrial dysfunction and impaired autophagy [30]. Our results revealed higher expression of gasdermin D in sarcopenic muscle from DP, further suggesting a connection between impaired autophagy and NLRP3-dependent inflammation in the development of severe sarcopenia.

MYH3 is a myosin isoform that is primarily expressed during embryonic development. However, once an individual is born, it is replaced by mature myosins that are responsible for adult muscle contraction [63]. Interestingly, it has been observed that MYH3 is re-expressed during muscle regeneration for a period of 2–3 weeks after injury [64]. However, in cases where muscle degeneration occurs, constant levels of MYH3 expression have been observed. Notably, research has focused on Duchenne muscular dystrophy, a devastating muscle pathology [63]. This condition is caused by mutations in the gene encoding the dystrophin protein. Dystrophin is crucial for maintaining the strength, flexibility, and stability of skeletal muscle. Its deficiency hinders proper contraction and relaxation of the muscle tissue [63]. Consequently, dystrophic muscle exhibits higher levels of MYH3 expression due to its continuous and inadequate regeneration.

Hence, drawing from the observations made in Duchenne muscular dystrophy, we analyzed the expression of *MYH3* in our patients and found a pronounced upregulation in sarcopenic muscle from DP, together with a downregulation of the adult myosin isoform *MYH2*. These results show dead ends, unsuccessful attempts at muscle regeneration which lead to sarcopenia in elderly dependents. Consistently with the findings in dystrophic muscle of *mdx* mice [65], we found a sharp upregulation of the *MYH3*/*MYH2* ratio in dependent elderly with sarcopenia indicating a shift in myosin isoform expression from the mature to the embryonic form, which recapitulates a feature of DMD. Consequently, it is plausible that the affected tissue undergoes continuous damage and inconclusive regeneration. The higher expression of MYH3, in conjunction with the absence of activation of myogenic precursors typically found in healthy regenerating muscle in individuals with sarcopenia [46], signifies a pathological process of incomplete regeneration that may result in the atrophy of aging muscles. Therefore, the higher expression of the embryonic myosin 3 can serve as a valuable indicator of muscle damage in previously unidentified degenerative disorders affecting human muscle. These findings support the use of *MYH3* gene expression or the *MYH3*/*MYH2* ratio as a marker of sarcopenia worsening.

Moreover, upregulation of NLRP3 was shown in DMD with higher Caspase-1 activity and larger levels of mature IL-1β and IL-18 [66]. NLRP3 knock-out in mdx mice led to a reduction in inflammation and oxidative stress in dystrophic muscle and resulted in a higher global muscle force attenuating the dystrophic phenotype. These findings, in concordance with our data, indicate that the NLRP3 inflammasome plays a crucial role in muscle inflammatory processes and related myopathies, highlighting its potential as a therapeutic target.

Sarcopenia not only impacts muscle tissue, but also initiates a sequence of systemic modifications. Specifically, it has been documented that this geriatric syndrome is closely linked to a state of persistent inflammation at the systemic level [67]. This persistent inflammation state triggers a range of modifications in multiple blood components, with particular significance at the level of erythrocytes. The surplus of pro-inflammatory cytokines hampers the production of erythropoietin, a protein responsible for the maturation and proliferation of red blood cells [68,69]. Consequently, the red blood cell distribution width (RDW) is significantly altered. Chronic inflammation has been observed to elevate RDW levels [68] and is now recognized as an indicator of an excessive inflammatory response [70]. Hence, this study evaluated the resistance of erythrocytes to rupture or destruction by employing the hemolysis test. The findings revealed that elderly individuals with dependency exhibit a higher rate of erythrocyte hemolysis compared to independent elderly individuals. This suggest that elderly individuals with severe sarcopenia experience a higher vulnerability of their erythrocytes to damage and premature destruction under specific adverse conditions, such as chronic inflammation and increased oxidative stress. Consequently, the findings from the current investigation, which indicate an activation of the inflammatory response, align with the results of a prior study on dependent elderly individuals that observed a significant increase in oxidative damage [30]. Collectively, these findings support the notion that severe sarcopenia creates an inflammatory unfavorable environment that may contribute to an accelerated destruction of red blood cells, thereby reducing their lifespan.

Inflammaging, an enduring condition of inflammation distinguished by increased levels of inflammatory markers in the blood, is closely linked to various age-related ailments such as sarcopenia [9]. This chronic inflammation not only increases the risk of chronic diseases but also contributes to disability and frailty in individuals. Senescence-associated secretory phenotype (SASP) constitutes a maladaptive response driving to inflammaging, aging phenotypes, and pathologies late in life. The SASP factors can be divided into the following major categories: soluble signaling factors, which include interleukins, chemokines, and growth factors; secreted proteases and secreted insoluble proteins/extracellular matrix (ECM) components [71]. In this study, we found that individuals diagnosed with dependency exhibit a pro-inflammatory phenotype characterized by the increased expression of Caspase-1, IL-6, IL-1ß, as well as an elevation in the presence of CD68^+^ inflammatory cells. The senescence secretory profile plays a pivotal role in the initiation and advancement of various myopathies [72,73]. Additionally, we found high levels of protein expression of cell-cycle inhibitor p16^INK4a^ indicating an increase in senescence cells in sarcopenic muscle from DP. In turn, p16 activates the NLRP3 inflammasome pathway by increasing integrin alpha L (ITGAL) and integrin alpha M (ITGAM) expression [74] making a feedback loop [75].

RIP3 has been reported to play a role in the activation of the NLRP3 inflammasome and infiltrating macrophages during acute lung injury [41], features we also identified in sarcopenic muscle from DP with increased expression of RIP3 in skeletal muscle and increase in cell trafficking of macrophages and leucocytes present in the tissue. Furthermore, RIP3-dependent activation of the NLRP3 inflammasome pathway has been found in inflammatory diseases such as lupus nephritis [76] and early brain injury following subarachnoid hemorrhage [77].

The NLRP3 inflammasome has been observed to impact the DNA damage responses following oxidative stress in dendritic cells [78]. Additionally, it has been found to activate the NLRP3 inflammasome in keratinocytes in response to nuclear DNA damage [79], suggesting the presence of a potential activation feedback loop. Although not statistically significant, our findings indicate a rise in protein levels of p-H2AX, indicating a tendency towards higher levels of DNA damage in sarcopenic muscle from DP compared to IP.

Extracellular ATP serves as a danger signal to alert the immune system and behave as an inflammatory cue acting as an intercellular messenger [80]. ATP has been characterized as an activator of the inflammasomes, which in turn triggers inflammation in the heart and leads to cardiac hypertrophy through the activation of NLRP3 [18]. Likewise, the presence of free plasma ATP has been identified as a factor that activates NLRP3 in dental pulp fibroblast in an ROS-dependent manner [81]. Mitochondrial alterations that result in increased ROS and subsequent cellular oxidative damage have been observed in patients with dependency [30] and these changes may explain the increased ATP we detected in the same group.

Aberrant inflammasome signaling has been associated with the progression of numerous conditions, including cardiovascular and metabolic diseases, cancer, and neurodegenerative disorders. Recently, inflammasomes have been proposed as potential targets for therapeutic interventions in human diseases. These findings present an opportunity for various clinical intervention strategies aimed at reducing the inflammatory phenotype. Firstly, the application of senolytic medications can be considered to hinder the release of DAMPs from senescent cells, thereby preventing the activation of the immune response. Alternatively, another approach involves directly targeting the NLRP3 inflammasome using inhibitors.

**Figure 7:**
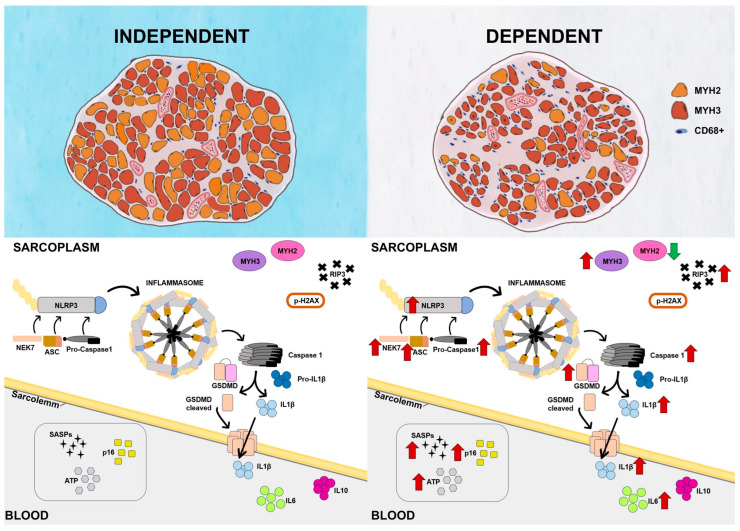
Overview of NLRP3 inflammasome pathway in skeletal muscle of independent and depenedent elderly patients.

Taken together, our findings support the idea that the NLRP3 inflammasome becomes active in human sarcopenic muscle from DP through the upregulation of the ASC, NEK7, Gasdermin D pathway (Figure 7). This activation leads to the activation of Caspase-1 and subsequently triggers the release of IL-1ß, which mediates the generation of a pro-inflammatory SASP. Additionally, the NLRP3 inflammatory phenotype may contribute to the process of injury and incomplete regeneration observed in the sarcopenic muscle from DP, as compared to the IP muscle. Finally, we identified the gene expression of MYH3 and the MYH3/MYH2 ratio as potential biomarkers of sarcopenia worsening.

## 4. Material and Methods

### 4.1. Experimental Design

The study population (n = 60; 53 women and 7 men; mean age 85.15 ± 5.82 years) belongs to the HIPA cohort and included a northwest Spanish cohort of older (≥70 years) individuals who were undergoing hip fracture surgery at Monte Naranco Hospital (Oviedo, Spain). To assess the population’s level of independence in performing daily activities, we employed the Barthel Index (BI) as a measure of their functional capacity. 

A cohort of 86 healthy patients was selected for this study, and both blood samples and skeletal muscle biopsies were collected from each participant. Among these individuals, 30 were classified as functionally independent with a Barthel Index (BI) score of 100, while the remaining 30 patients were categorized as functionally dependent with a BI score below 70 (Supplementary Table S1). The inclusion of both groups aimed to examine the association between physical disability, as reflected by low BI levels, and the concurrent muscle loss, which is a defining characteristic of extreme sarcopenia. The exclusion criteria were pathologic fracture, severe cognitive impairment, premorbid obesity (BMI > 30), and being in a terminal stage of a disease or with rapidly fatal underlying disease and estimated lifespan of less than 6 months (Supplementary Table S2). Each patient signed written informed consent. The current study was performed according to the Declaration of Helsinki and was approved by all national and local ethical committees (REF. 76/2013).

### 4.2. Biochemical Blood Analysis

Blood samples were collected from patients through venipuncture in the morning following an overnight fasting period and a 15 min rest. The biochemical analysis of the blood was conducted using an automated FALCOR 560 analyzer (Menarini Diagnostics, Barcelona, Spain) in a highly standardized hematology and biochemical laboratory at Monte Naranco Hospital. To assess the hemolysis of erythrocyte cells, a modified technique developed by Farrell and colleagues [82] was employed. The protocol described by de Gonzalo-Calvo and colleagues [83] was followed for this procedure. Finally, the outcomes were expressed as a percentage of the total hemoglobin released from erythrocytes that were diluted in distilled water.

### 4.3. Muscle Collection and Homogenization

Skeletal muscle samples from the *vastus lateralis* were immersed and transported in HBSS with 1% Penicillin-Streptomycin (15240-062, Thermo Scientific, Waltham, MA, USA) from the surgery room and were immediately processed, frozen in liquid nitrogen, and stored at −80 °C. Muscle samples were homogenized using an Ultra-turrax T25 homogenizer (Staufen, Germany) in a proportion of 200 mg tissue/500 μL of lysis buffer (50 mM phosphate-sodium buffer pH 7.5, 1 mM PMSF, 1 mM NaF, 1 mM Na_3_VO_4_). Following, tissue homogenates were centrifuged at 1200 g at 4 °C for 5 min and lipid droplets and debris were removed. Final protein amount was estimated by Bradford method.

### 4.4. Gene Expression

The total muscle RNA extraction was isolated using TRI reagent (T9424, Sigma-Aldrich, Burlington, MA, USA) method. Purified RNA was reverse transcribed using Hiscript III 1st Strand cDNA Synthesis Kit (+gDNA Wiper) (NB-54-0182-02, NeoBiotech, France).

Quantitative RT-PCR gene expression was performed using StepOnePlus (Applied Biosystems, MA, USA) and following the TB Green Premix Ex Taq II (Tli RNaseH Plus) (RR820A, Takara Bio Inc., Shiga, Japan) manufacturer’s protocol. The expression of six candidate genes was evaluated: NLRP3, ASC, NEK7, GSDMD, MYH2 (myosin type 2A), and MYH3 (myosin type 3). HPRT1 gene expression was used as endogenous control. Specific primers to each gene were designed by our laboratory and its sequences and references can be found in Supplementary Table S4.

### 4.5. Enzyme-Linked Immunosorbent Assay 

Interleukin 6 (IL-6) (KMC0061, Life Technologies, CA, USA), IL-10 (D1000B, R&D Systems, MN, USA) and IL-1 beta (A270338, Antibodies, Bromma, Sweden) ELISA kits were used to characterize the inflammatory response in plasma samples. All ELISA assays were performed according to manufacturer’s protocols.

### 4.6. Caspase-1 Activity Measurement

Caspase-1 activity was measured in tissue samples using Caspase-Glo^®^ 1 Inflammasome Assay (G9951, Promega, Southampton, UK). The assay was performed in a 1:1 ratio of Caspase-Glo^®^ 1 reagent to muscle protein lysate. The mix was incubated at room temperature for 2 h and the luminescent reaction was measured on a SIRIUS luminometer (Berthold, Pforzheim, Germany).

### 4.7. Adenosine 5′-Triphophate Measurement

Plasma and erythrocytes ATP levels were evaluated using the ATP Bioluminescent Assay Kit (FLAA, Sigma-Aldrich, Burlington, MA, USA). ATP measurement is based on its consumption when firefly luciferase catalyzes the oxidation of D-luciferin. The assay was assessed following the manufacturer’s recommendation and ATP content was determined by the light emission enhanced by the oxidation reaction using SIRIUS luminometer (Berthold, Pforzheim, Germany).

### 4.8. Western Blotting

Western blotting immunoassays were performed following the protocol described by previous work of our research group [50]. Primary antibodies were incubated at 4 °C overnight; meanwhile, the secondary antibodies were incubated at room temperature for 1 h. All the additional antibodies’ information is indicated in Supplementary Table S5. The GeneTools 4.3.17.0 (Syngene, Cambridge, UK) allowed us to quantify the optical density of the bands. The results of the densitometry were normalized to Ponceau Staining (P3504, Merck) as loading of total protein control. Ponceau S were used due to the classic housekeeping protein levels (as GAPDH, β-Actin and α-Tubulin) showing differences between samples.

### 4.9. Creatine Kinase Assay

Muscle CK activity was evaluated in muscle lysates using Creatine Kinase Activity Assay Kit (MAK116, Sigma-Aldrich, Burlington, MA, USA) according to the manufacturer’s protocol. Upon the addition of 10µL of samples, the absorbance of CK activity was measured spectrophotometrically at 340 nm with Biotek PowerWave XS Microplate Reader (Marshall Scientific, Hampton, NH, USA). Absorption values were normalized to creatine kinase standard curve and expressed as activity units/L.

### 4.10. Histology and Immunostainings

Skeletal muscle biopsies were fixed in paraformaldehyde (PFA) 4% at 4 °C for 2 h; washed with PBS at 4 °C; and cryoprotected with sucrose 15% at 4 °C for 72 h. Cryoprotected samples were mounted in OCT (4583, TissueTek, Sakura Finetek Europe, The Netherland) in plastic cryomolds before being snap frozen in cooled isopentane using liquid nitrogen and subsequently stored at −80 °C. After that, muscle was sectioned with cryostat at −20 °C (10 µm cryosections) and mounted on Superfrost plus-coated slides (J1800AMNZ, Thermo Scientific, Waltham, MA, USA).

After reaching room temperature, slides were fixed with PFA 4% at room temperature for 10 min. Sample permeabilization and blocking was performed in PBS with 0.1% Triton-100X and Normal Donkey Serum 10% at room temperature for 1 h. Processed slides were incubated with anti-Laminin (Sigma L93993, 1:100), anti-CD68 (Dako Clone PG-M, neat), and anti-CD45 (Dako Clones 2B11 + PD7/26, neat) primary antibodies at 4 °C overnight. Slides were incubated with secondary antibodies (Alexa 488 and Alexa 555) diluted in PBS at room temperature for 1 h. Coverslips were mounted with Vectashield Antifade Mounting Medium with DAPI (H-1200, Vector Labs, Newark, CA, USA). Delphi-X Inverso microscope DI.3053-PLPHF (Euromex, Arnhem, The Netherlands) was used to obtain images. Image analysis was performed with Fiji Software using the “cell counter” package. Leica SP8 confocal microscope (Leica Microsystems, Wetzlar, Germany) was used to show average representative images.

### 4.11. Statistical Analysis

Data are represented as the mean ± standard error of the mean (SEM). The normality of the data was verified using the Kolmogorov–Smirnov test. If the parameters adhere to the assumption of normality, we conducted a Student’s t-test on paired samples to compare the two experimental groups. However, if the parameters do not meet the normality assumption, we employed the Mann–Whitney test to compare the variables between the groups. Differences were considered statistically significant with a *p* < 0.05. Asterisks indicate level of statistical significance: * *p* ≤ 0.05, ** *p* ≤ 0.01, *** *p* ≤ 0.001, **** *p* ≤ 0.0001. Statistical and graphical analyses were performed with GraphPad Prism 6.0. 

## Data Availability

Data is contained within the article and supplementary material.

## Supplementary Materials

The following supporting information can be downloaded at: https://www.mdpi.com/article/10.3390/ijms25031439/s1.
