# Supplementary material for: NLRP3 Contributes to Sarcopenia Associated to Dependency Recapitulating Inflammatory-Associated Muscle Degeneration
Source: Int J Mol Sci. 2024 Jan 24;25(3):1439. doi: 10.3390/ijms25031439 (PMC10855188; doi:10.3390/ijms25031439)
Supplement: Supplementary file 1 [file ijms-25-01439-s001.zip › ijms-2811815-supplementary.pdf]

Table S1. Study participant characteristics.

	Independent	Dependent	*P*-value	Significance
N	30	30	-	-
Age	84 ± 6	86 ± 5	0.1058	N.S.
Sex (women)	27 (90%)	26 (86.67%)	>0.9999	N.S.
Bathel Index	100	38.5 ± 17.28	<0.0001	****
Charlson Index	1.200 ± 1.157	1.900 ± 1.583	0.0794	N.S.

Data are represented as the mean ± SD. N.S., not significant. **** P<0.0001

Table S2. Patients background information

Pathologie	Independent	Dependent	P-value	Significance
Hypertension	24 (80%)	20 (66.67%)	0.3817	N.S.
Diabetes	5 (16.67%)	7 (23.33%)	0.7480	N.S.
Dyslipidemia	10 (33.33%)	13 (43.33%)	0.5959	N.S.
Cardiac insufficiency	2 (6.67%)	2 (6.67%)	>0.9999	N.S.
Ischemic Cardiopathy (IC)	9 (30%)	6 (20%)	0.5520	N.S.
Stroke	6 (20%)	6 (20%)	>0.9999	N.S.
Chronic Obstructive Pulmonary Disease (COPD)	0 (0%)	3 (10%)	0.2375	N.S.
Mild-moderate dementia	3 (10%)	18 (60%)	<0.0001	****
Creatinine (md/dL)	0.912 ± 0.0468	1.145 ± 0.1699	0.7325	N.S.
Chronic Kidney Disease (CKD)	0 (0%)	3 (10%)	0.2375	N.S.
Cancer	6 (20%)	8 (26.67%)	0.7611	N.S.

Data are represented as the mean ±SEM. N.S., not significant. **** P<0.0001

Table S3. White blood cell (WBC) count and red blood cell (RBC).

	Independent	Dependent	Units	P-value	Significance
Leucocites	8.526 ± 0.583	9.331 ± 0.826	*10e3/uL	0.3678	N.S.
Neutrophils	6.415 ± 0.5707	7.257 ± 0.805	*10e3/uL	0.5205	N.S.
Linfocites	1.188 ± 0.092	1.132 ± 0.091	*10e3/uL	0.6703	N.S.
Monocites	0.747 ± 0.060	0.731 ± 0.068	*10e3/uL	0.5203	N.S.
Eosinophils	0.145 ± 0.027	0.189 ± 0.028	*10e3/uL	0.1448	N.S.
Basophils	0.029 ± 0.003	0.032 ± 0.004	*10e3/uL	0.9226	N.S.
Erytrocites	3.8 ± 0.089	15.71 ± 11.98	*10e6/uL	0.7832	N.S.

Data are represented as mean ± SEM. N.S., not significant.

Table S4. List of primers and reference genes

Name	Forward	Reverse	Reference Ensenbl	Reference NM
NLRP3	CGAGGGGTCAGACAGAGAAG	TTCCTGGCATATCACAGTGG	ENST00000336119.8	NM_001243133.2
ASC	AAGCCAGGCCTGCACTTTAT	CAGGCTGGTGTGAAACTGAA	ENST00000247470.10	NM_013258.5
GSDMD	GCTCCAGCACCTCAATGAAT	GACCGTCTTCTTCTGGCTCA	ENST00000262580.9	NM_024736.7
NEK7	AGCAATCACAAGGAATGCAAG	GGTACTCCATCCAAGAGACAGG	ENST00000367385.9	NM_133494.3
MYH2	GATAAGATCCTGGCAGAATGGA	TCTGCAATCTGTTCCGTGAG	ENST00000245503.10	NM_017534.6
MYH3	ACAGACAGCGCCATTGACA	AGGCTGTTTTGTCAGCCACT	ENST00000583535.6	NM_002470.4
HPRT1	ATGGACTGATTATGGACAGGACTG	TCCAGCAGGTCAGCAAAGAAC	ENST00000298556.8	NM_000194.3

Table S5. Antibody table. Details of sources of antibodies used for Western blotting.

Antibody	Manufacturer	Reference
Anti Phospho-Histone H2A.X (Ser139)	Cell Signaling	#9718
Anti p16 INK4A (F-12)	Santa Cruz	sc-1661
Anti RIP3 (E7A7F) XP	Cell Signaling	#10188
Anti Rabbit IgG, HRP	Cell Signaling	#7074
Anti Mouse IgG, HRP	Cell Signaling	#7076
